# Supplementary figures and images for: Ambient air pollution exposure and effects on neutralizing antibody titers following SARS-CoV-2 vaccination in adults
Source: PLOS Glob Public Health. 2025 May 12;5(5):e0004609. doi: 10.1371/journal.pgph.0004609 (PMC12068591; doi:10.1371/journal.pgph.0004609)

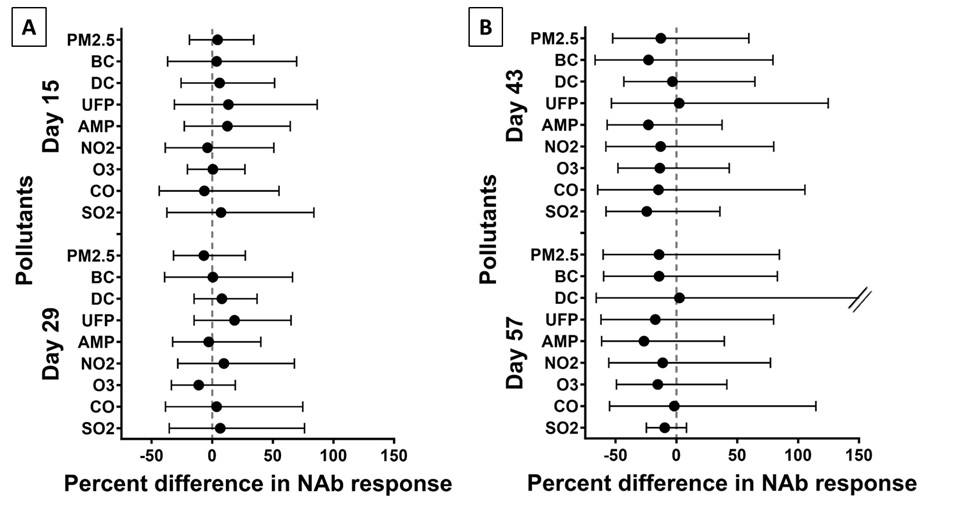

Supplement: S1 Fig — Of note, the upper CI for DC at day 57 extends past the end of the graph (denoted by a double forward slash). (TIF) [file pgph.0004609.s001.tif]

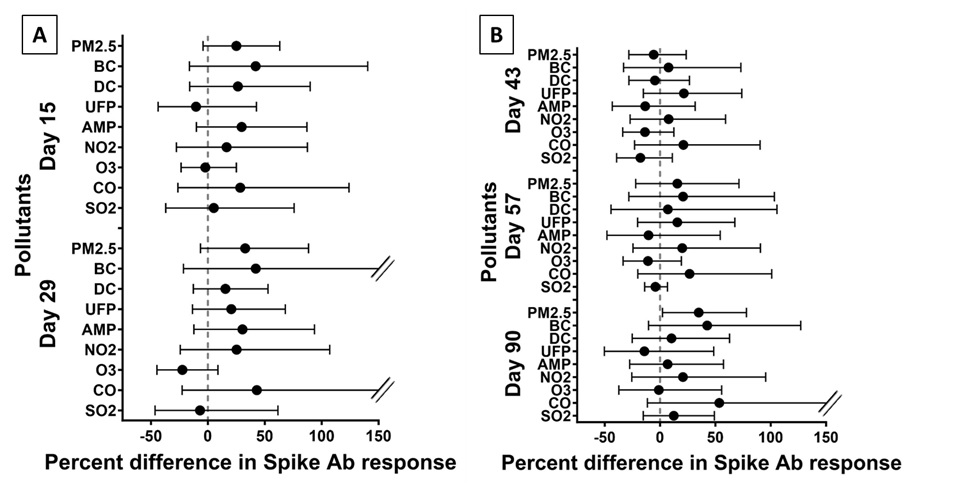

Supplement: S2 Fig — Of note, the upper CI for BC at day 29 and CO at days 29 and 90 extends past the end of the graph (denoted by a double forward slash). (TIF) [file pgph.0004609.s002.tif]
