# Supplementary material for: Ambient air pollution exposure and effects on neutralizing antibody titers following SARS-CoV-2 vaccination in adults
Source: PLOS Glob Public Health. 2025 May 12;5(5):e0004609. doi: 10.1371/journal.pgph.0004609 (PMC12068591; doi:10.1371/journal.pgph.0004609)
Supplement: S1 Text — (DOCX) [file pgph.0004609.s003.docx]

### **Ambient air pollution exposure and effects on neutralizing antibody titers following SARS-CoV-2 vaccination in adults**

Daniel P. Croft,^1,2*^Carl J. Johnston,^3^ Angela R. Branche,^4^ David Q. Rich,^1,2,5^ Philip K. Hopke,^2,5,6^ Kelly Thevenet-Morrison,^5^ Sally. W. Thurston,^2,7^ Todd A. Jusko,^2,3,5^ Md Rayhanul Islam,^5^ Catherine Bunce,^4^ Michael C. Keefer,^4^ Edward E. Walsh^4^ and Ann R. Falsey^4^

^1^ Department of Medicine, Division of Pulmonary and Critical Care Medicine, University of Rochester, Rochester, New York, United States of America

^2^ Department of Environmental Medicine, University of Rochester Medical Center, Rochester, New York, United States of America

^3^ Department of Pediatrics, University of Rochester. Rochester, New York, United States of America

^4^ Division of Infectious Disease, Department of Medicine, University of Rochester, Rochester, New York, United States of America

^5^ Department of Public Health Sciences, University of Rochester, Rochester, New York, United States of America

^6^ Institute for a Sustainable Environment, Clarkson University, Potsdam, New York, USA

^7^ Department of Biostatistics and Computational Biology, University of Rochester, Rochester, New York, United States of America

*Corresponding author:

E-mail: [daniel_croft@urmc.rochester.edu](mailto:daniel_croft@urmc.rochester.edu)

**IQR discussion:**

A linear regression provides the difference in the outcome per one unit increase in pollutant increase. If we want to compare the size of effects across pollutants though, we cannot validly do that with a 1-unit increase. PM2.5, BC, and DC are in units of µg/m^3^, while UFP is in units of particles/cm^3^. Therefore a 1 unit increase in UFP is a much smaller exposure than a 1 unit increase in µg/m^3^. Therefore, we could falsely conclude that DC is much more important than UFP in causing a change in NAb simply because the size change in NAb per 1 unit increase in DC (e.g., 20%) looks much larger than the size change in NAb per 1 unit increase in UFP (e.g., 1%), even though they are both statistically significant. Therefore, to make a valid comparison between pollutants, we must choose some facet reflecting he distribution of the pollutant observed during the study time. We could use a standard deviation, 5^th^ to 95^th^ percentile, or a 25^th^-75^th^ percentile change (interquartile range). Many have settled on using the IQR for this purpose. This does not change any of the hypothesis test, nor whether it is a precise or imprecise effect estimate. This is just a ‘scaling’ factor to allow valid comparisons across pollutants.
