# Supplementary material for: Ambient air pollution exposure and effects on neutralizing antibody titers following SARS-CoV-2 vaccination in adults
Source: PLOS Glob Public Health. 2025 May 12;5(5):e0004609. doi: 10.1371/journal.pgph.0004609 (PMC12068591; doi:10.1371/journal.pgph.0004609)
Supplement: Attachment — Submitted filename: Response to reviewers_Vaccineairpollution_Croft.docx [file pgph.0004609.s005.docx]

PGPH-D-24-00744
Ambient air pollution exposure and effects on neutralizing antibody titers following SARS-CoV-2 vaccination in adults
PLOS Global Public Health

Dear Colleagues,

Thank you for reviewing our manuscript. Please find our responses to your thoughtful comments below. Please note that line numbers refer to the tracked changes version with ‘All markup’ selected.

Sincerely,

Daniel Croft MD, MPH, ATSF

Associate Professor

Dept of Medicine: Pulmonary and Critical Care Division

Dept of Environmental Medicine

University of Rochester Medical Center

*PLOS Global Public Health*

*Journal Requirements:*

1. *Please send a completed 'Competing Interests' statement, including any COIs declared by your co-authors. If you have no competing interests to declare, please state "The authors have declared that no competing interests exist". Otherwise please declare all competing interests beginning with the statement "I have read the journal's policy and the authors of this manuscript have the following competing interests:"*

**Author response:** We have included this statement in the cover letter and in lines 369-375 of the revised manuscript (with track changes).

 “Dr. Angela Branche reports financial support including research funding from Moderna, Pfizer, Cyanvac and Vaccine.com. Dr. Branche has served on an advisory board for Novavax and speakers bureau for Moderna and GSK. Dr. Ann R. Falsey has received consulting fees and research grants from Merck Sharpe and Dohme, Pfizer, Astra Zeneca, Novavax, Janssen, CyanVac, Vax Co, Moderna, Sanofi Pasteur, GSK and BioFire Diagnostics. Dr. Edward E. Walsh reports grants from Merck and Pfizer Inc, served as a consultant to Moderna, Merck, GlaxoSmithKline, and Janssen. All other authors declare that they have no known competing financial interests or personal relationships that could have appeared to influence the work reported in this paper.”

*2. Please amend your detailed Financial Disclosure statement. This is published with the article. It must therefore be completed in full sentences and contain the exact wording you wish to be published.*

*****Please only choose the relevant sentences from below*****

*1. Please clarify all sources of funding (financial or material support) for your study. List the grants (with grant number) or organizations (with url) that supported your study, including funding received from your institution.*

*2. State the initials, alongside each funding source, of each author to receive each grant.*

*3. State what role the funders took in the study. If the funders had no role in your study, please state: “The funders had no role in study design, data collection and analysis, decision to publish, or preparation of the manuscript.”*

*4. If any authors received a salary from any of your funders, please state which authors and which funders.*

*If you did not receive any funding for this study, please simply state: “The authors received no specific funding for this work.”*

**Author response:** We have edited the financial disclosure.

*3. In the online submission form, you indicated that " As the proprietary data on neutralizing antibody and spike protein IgG were obtained from Astra Zeneca for this study, data requests for NAb and spike protein IgG will need to be addressed to Astra Zeneca for permission to use these data.".*

*All PLOS journals now require all data underlying the findings described in their manuscript to be freely available to other researchers, either*

*1. In a public repository,*

*2. Within the manuscript itself, or*

*3. Uploaded as supplementary information.*

*This policy applies to all data except where public deposition would breach compliance with the protocol approved by your research ethics board. If your data cannot be made publicly available for ethical or legal reasons (e.g., public availability would compromise patient privacy), please explain your reasons by return email and your exemption request will be escalated to the editor for approval. Your exemption request will be handled independently and will not hold up the peer review process, but will need to be resolved should your manuscript be accepted for publication. One of the Editorial team will then be in touch if there are any issues.*

**Author response:** We have responded by email to this request.

*4. We do not publish any copyright or trademark symbols that usually accompany proprietary names, eg (R), (C), or TM  (e.g. next to drug or reagent names). Please remove all instances of trademark/copyright symbols throughout the text, including "Meso Scale Discovery®" on page*

**Author response:** As suggested, we have removed trademark/copyright symbols.

*5. Please provide an Author Summary. This should appear in your manuscript between the Abstract (if applicable) and the Introduction, and should be 150–200 words long. The aim should be to make your findings accessible to a wide audience that includes both scientists and non-scientists. Sample summaries can be found on our website under Submission Guidelines:*

[*https://journals.plos.org/globalpublichealth/s/submission-guidelines#loc-parts-of-a-submission*](https://urldefense.com/v3/__https:/journals.plos.org/globalpublichealth/s/submission-guidelines*loc-parts-of-a-submission__;Iw!!MkQhnT5yHH0MPvOc00c!Xs1LgmE2i8AtIIpO07Q2gZzY9x45TD-QYGT_JVG0V7BVjcqjooKSlkm8qUy_TP285Xc61QCnA-WqUUukKrNXN4wRHq_o$)

**Author response:** As suggested we have added an author summary to the manuscript (lines 50-61):

The majority of the world breathes outdoor PM_2.5_ concentrations above the WHO standard. COVID-19 vaccines protect individuals by causing an immune response leading to increased Neutralizing antibodies (NAb) to a selected antigen called spike protein from the SARS-CoV-2 virus. Past studies have observed changes in the immune response to vaccines related to environmental exposures. In our pilot study we matched the 14 days of pollution prior to each blood draw in a group of 74 patients who received a specific COVID-19 vaccine (AZD1222). In part due to our small sample size, our results did not reach statistical significance and had high variability. Though imprecise, we observed that PM_2.5_ and other pollutants generally showed a pattern of decreased NAb titers in the time period after the second AZD1222 vaccine. The fact that Rochester, NY is a low pollution area makes our study policy relevant as our PM_2.5_ concentrations are below the National Ambient Air Quality Standards. While our study will need to be replicated a larger multicenter study, if air pollution reduces immunity from AZD1222, vaccine dosing or frequency may need adjustment to maximize efficacy according to pollution exposure.

*6. "AZ vaccine and air pollution (1).tif": Please confirm whether you drew the images / clip-art within the figure panels by hand. If you did not draw the images, please provide (a) a link to the source of the images or icons and their license / terms of use; or (b) written permission from the copyright holder to publish the images or icons under our CC-BY 4.0 license. Alternatively, you may replace the images with open source alternatives. See these open source resources you may use to replace images / clip-art:*

**Author response**: all contents of the graphical abstract figure were created by the authors with biorender software. The license for publication of this figure is attached to this submission.

*-* [*https://commons.wikimedia.org*](https://urldefense.com/v3/__https:/commons.wikimedia.org__;!!MkQhnT5yHH0MPvOc00c!Xs1LgmE2i8AtIIpO07Q2gZzY9x45TD-QYGT_JVG0V7BVjcqjooKSlkm8qUy_TP285Xc61QCnA-WqUUukKrNXNxvtzcWP$)

*-* [*https://openclipart.org/*](https://urldefense.com/v3/__https:/openclipart.org/__;!!MkQhnT5yHH0MPvOc00c!Xs1LgmE2i8AtIIpO07Q2gZzY9x45TD-QYGT_JVG0V7BVjcqjooKSlkm8qUy_TP285Xc61QCnA-WqUUukKrNXN3OmMW3x$)

*Additional Editor Comments (if provided):


[Note: HTML markup is below. Please do not edit.]

Reviewers' comments:

Reviewer's Responses to Questions*

***Comments to the Author*** *1. Does this manuscript meet PLOS Global Public Health’s* [*publication criteria*](https://urldefense.com/v3/__https:/track.editorialmanager.com/CL0/https:*2F*2Fjournals.plos.org*2Fglobalpublichealth*2Fs*2Fcriteria-for-publication/1/010f0193f1dcfaa6-da46ce0a-6b1b-4ece-8727-7d7159d78541-000000/WFT7LnnxkR_shTT4hxjONmbvVO2N6cg_4aBwUqcQveI=190__;JSUlJSU!!MkQhnT5yHH0MPvOc00c!Xs1LgmE2i8AtIIpO07Q2gZzY9x45TD-QYGT_JVG0V7BVjcqjooKSlkm8qUy_TP285Xc61QCnA-WqUUukKrNXNyDjvIlD$)*? Is the manuscript technically sound, and do the data support the conclusions? The manuscript must describe methodologically and ethically rigorous research with conclusions that are appropriately drawn based on the data presented.*

*Reviewer #1: Partly*

*Reviewer #2: Partly*

*2. Has the statistical analysis been performed appropriately and rigorously?*

*Reviewer #1: Yes*

*Reviewer #2: No*

*3. Have the authors made all data underlying the findings in their manuscript fully available (please refer to the Data Availability Statement at the start of the manuscript PDF file)?

The* [*PLOS Data policy*](https://urldefense.com/v3/__https:/track.editorialmanager.com/CL0/https:*2F*2Fjournals.plos.org*2Fglobalpublichealth*2Fs*2Fdata-availability/1/010f0193f1dcfaa6-da46ce0a-6b1b-4ece-8727-7d7159d78541-000000/mwNwf_r3JDve4G6vUxop1Tmr8-r8V4fRZ4WegqtEXeE=190__;JSUlJSU!!MkQhnT5yHH0MPvOc00c!Xs1LgmE2i8AtIIpO07Q2gZzY9x45TD-QYGT_JVG0V7BVjcqjooKSlkm8qUy_TP285Xc61QCnA-WqUUukKrNXN26Azmxu$) *requires authors to make all data underlying the findings described in their manuscript fully available without restriction, with rare exception. The data should be provided as part of the manuscript or its supporting information, or deposited to a public repository. For example, in addition to summary statistics, the data points behind means, medians and variance measures should be available. If there are restrictions on publicly sharing data—e.g. participant privacy or use of data from a third party—those must be specified.*

*Reviewer #1: Yes*

*Reviewer #2: No*

*4. Is the manuscript presented in an intelligible fashion and written in standard English?

PLOS Global Public Health does not copyedit accepted manuscripts, so the language in submitted articles must be clear, correct, and unambiguous. Any typographical or grammatical errors should be corrected at revision, so please note any specific errors here.*

*Reviewer #1: Yes*

*Reviewer #2: Yes*

*5. Review Comments to the Author

Please use the space provided to explain your answers to the questions above. You may also include additional comments for the author, including concerns about dual publication, research ethics, or publication ethics. (Please upload your review as an attachment if it exceeds 20,000 characters)*

*Reviewer #1: This interesting pilot study addresses an important research question: whether acute air pollution exposure impairs vaccine responses. If the data are confirmed in other settings, larger studies should be conducted to evaluate to what extent vaccination protects from the deleterious effects of air pollution on Covid incidence and severity.
The authors have made a great effort, but some aspects are unclear and need revaluation.*

*1. The authors need to explain why they did not perform the same analysis in the placebo group. The findings would have strengthened the results.*

**Author response**: We did consider adding the placebo group to this study. However, there was minimal change in neutralizing antibody or spike protein within the control group (Median of 20 for Neutralizing Ab at all time points from Falsey et al (2021) below). Given the lack of change in the Nab concentrations, we determined that the pairing of air pollution with this placebo group would not contribute to answering our question of whether air pollution would be associated with a blunting of the antibody response to COVID vaccination. We agree that the placebo group could have use in answering a separate question of whether there an antibody response to air pollution in general (although vaccine status would need to be controlled for in that hypothetical study). Finally, the placebo group were not consented for our present air pollution study as they did not directly contribute to the primary research question. In summary, the placebo group were not included in the analysis. We edited the following line in the manuscript (lines 120-122):

“As this present study focused on the effect of air pollution on immune response to vaccination, only participants who received the AZD1222 vaccine were included (not individuals in the placebo arm).

Supplemental figure from Falsey et al (2021).

Falsey, A. R., Sobieszczyk, M. E., Hirsch, I., Sproule, S., Robb, M. L., Corey, L., ... & Gonzalez-Lopez, A. (2021). Phase 3 safety and efficacy of AZD1222 (ChAdOx1 nCoV-19) Covid-19 vaccine. *New England Journal of Medicine*, *385*(25), 2348-2360.

*2. Temporal confounding may be an issue, as the authors did not control for that. Temporal changes in air pollution were possibly related to changes in viral circulation (which could affect the circulating levels of antibodies).*

**Author response:** We agree that temporal confounding is an issue to consider due to pandemic dynamics due to COVID-19. As for societal restrictions, New York State lifted formal restrictions on June 15th of 2020, preceding enrollment through this trial. As for viral circulation, we assume that circulating viral load would be negatively correlated with air pollution as societal shutdown due to increased burden of infection in the community (increased viral circulation) would result in decreased pollution due to behavioral modifications or restrictions (decreased car/plane travel). It could also be assumed that higher circulating virus would lead to increase baseline antibodies in the general population. Therefore, if we did not control for the confounding effects of viral circulation, we may have underestimated the magnitude of the decrease in antibody response to PM_2.5_ (and other pollutants). However, this underestimation may be minimal as the participants took particular care to avoid infection during the study. Ultimately it is not likely that this aspect of temporal confounding made a substantial difference in our results. As suggested, we have added additional text to the limitations lines 349-355.

“Fourthly, we recognize that temporal confounding related to pandemic dynamics including viral circulation, changes in air pollution, and other behavioral changes in the population secondary to the COVID-19 pandemic were likely present. Specifically, viral circulation (community infection) would likely be correlated with decreased pollution (decreased car/plane travel) and increased Neutralizing antibody levels in participants if infected. This would theoretically lead to an underestimation of the magnitude of the decreased antibody response to PM2.5 (and other pollutants). Though ultimately, the risk of confounding from viral circulation was minimized by the cautious distancing approach undertaken by participants to avoid respiratory infections during the clinical trial minimized their risk of COVID-19 infection.”

*Minor comments.
The authors say in the introduction that the vaccination started in the winter 2020. I think it started later for the general population and this should be corrected.*

**Author response:** We have added another reference (Watson et al 2022) for the start of global vaccines (December 8, 2020 - NHS) and removed the comment on % vaccinated as it is less relevant at this point. Lines 77-78now read:

“Beginning in the winter of 2020, COVID-19 vaccines were recommended as a means of primary prevention.”

1. Watson, Oliver J et al. 2022 Global impact of the first year of COVID-19 vaccination: a mathematical modelling study. The Lancet Infectious Diseases, Volume 22, Issue 9, 1293 - 1302

*Reviewer #2: The linear mixed-effects model used for the statistical analysis is well-suited to the study design and data, however there are a few aspects that require further explanations. These aspects include the sample size, the adjustment of covariates, and the evaluation of effect significance, in this case non-significant statistical results. Regarding the results, the manuscript has to consistently communicate the non-statistically significant findings, particularly in the abstract and all related sections.
I also recommend the conclusion to state that no statistical significant effects were found. To be fair, there are such statements in the start of the Discussion and the conclusion says that association is "imprecise". This should be clear.*

**Author response:** As suggested we have added text in multiple locations including the abstract, discussion and conclusion to ensure that the reader understands that the results are not statistically significant. (Line 41, 238, 250, 295 and 362)

*The sample was limited to the number of participants and, more importantly, the number of residents within 15 miles of the air pollution measuring station. These restrictions limited the sample size to 74 individuals. What can you say about a minimum sample size regarding a few criteria? How much power can you establish from the sample size in the study?*

**Author response:** We agree that the small sample size was a key limitation to our pilot study. This study population is the only population available to us, so we did not have the ability to target a larger sample size based on a power calculation. In discussion with our biostatistics experts, a post hoc power calculation was not recommended. We were referred to the Heinsberg and Weeks article (2022) as an explanation of the limitations of these calculations outlined in the following quote“…post hoc power is misleading as it assumes that the observed effect size is similar to the true effect size, which can only be perfectly known when data are simulated. Finally, post hoc power is redundant with observed findings as there is a one-to-one relationship between the p-value and power.” Therefore we have opted not to include a discussion of power calculations in this article.

Heinsberg LW, Weeks DE. Post hoc power is not informative. Genet Epidemiol. 2022 Oct;46(7):390-394. doi: 10.1002/gepi.22464. Epub 2022 Jun 1. PMID: 35642557; PMCID: PMC9452450.

**doi:** [**10.1002/gepi.22464**](https://doi.org/10.1002/gepi.22464)

*Another significant limitation of the study is that all participants reside in the same city, which typically experiences low levels of air pollution. Also, if air pollution increases even slightly for any given moment during the study period, all individuals receiving the vaccine at the same time are exposed to the variation of same level of air pollution.
In fact, the descriptive analysis indicates that air pollutant concentrations remained very stable throughout the study, making it more challenging to assess any effects.*

**Author response:**

We agree that hypothetically if all vaccinated the same day this would be a major issue of temporal variability. However, the start dates for our patients were spread out over 15 different days over the course of 3 months. While it is true to that the small groups of participants who received the vaccine on the same day would have exposure to the same pollution values, the spread of start times over 3 months minimizes this concern. We included the following clarification on lines 357-359.

“Thirdly, we were also were mindful of the potential for batching of participants all experiencing the same pollution. However, with the start dates of the vaccination series spanning 3 months, we viewed this risk of loss of temporal variability as minimal.”

We agree that our location is in an area with mean PM_2.5_ concentrations below the national ambient air quality standards. Also, even increases in pollution similar to the magnitude of our study have been shown to associations with health effects in prior studies in Rochester NY (Yount et al 2023 and Hopke et al 2019 as two examples).

Yount CS, Utell MJ, Hopke PK, Thurston SW, Lin S, Ling FS, Chen Y, Chalupa D, Deng X, Rich DQ. Triggering of ST-elevation myocardial infarction by ultrafine particles in New York: Changes following Tier 3 vehicle introduction. Environ Res. 2023 Jan 1;216(Pt 1):114445. doi: 10.1016/j.envres.2022.114445. Epub 2022 Sep 29. PMID: 36181892.

Hopke, P. K., Croft, D., Zhang, W., Lin, S., Masiol, M., Squizzato, S., ... & Rich, D. Q. (2019). Changes in the acute response of respiratory diseases to PM2. 5 in New York State from 2005 to 2016. *Science of the total environment*, *677*, 328-339. https://doi.org/10.1016/j.scitotenv.2019.04.357

*I understand that variables sex and age were not included in all models due to not finding significant coefficients in the regression. However, these coefficients are not shown in the results.*

**Author response:**

We used proc glmselect with stepwise selection to evaluate whether a set of covariates were significant predictors of our outcomes, retaining only those covariates that were (i.e., p<0.05). As we had 90 models (multiple pollutants and multiple time points), we chose to use the proc covariate selection process followed by selection based on the frequency of significant associations between covariates and each outcome (NAb and Spike protein IgG). For example, we chose the covariate Sex for NAb as it had biologic plausibility and was the covariate with the highest frequency of significant associations with an outcome (i.e., in the 90 models of multiple pollutants/time points). However, these are just potential confounders, and thus results for these covariates not included in the results presented in the paper. We added the following text into the methods to clarify this point on lines 189-195.

“Of note, the linear regression method proc glmselect with stepwise selection was used for covariate selection, keeping only those covariates that were significant predictors of the outcome (p<0.05). The variables that we determined to be significantly associated with the outcomes were those covariates with the highest frequency of significant associations in these 90 models of multiple time points and multiple pollutants. Sex was the variable that was observed most frequently for models with NAb, and age was observed most frequently for models with spike protein IgG.”

*The methodology says that direction and magnitude of the effects were considered, rather than significance. I understand the reasoning about direction, but it is not possible to say much about direction/magnitude when uncertainty intervals are large, including positive and negative values. Therefore, the statistical significance has to play a role. This should be clear in the methodology.*

**Author response:** As suggested we have clarified the lack of statistical significance . We agree that the variability is high and there is no statistical significance to the results.

We have edited the methods to clarify this point on lines 199-203:

“While statistical significance was defined as a p value <0.05, we focused on the direction and magnitude of the effect estimates in this study to determine whether a consistent effect was observed between immune response outcomes (NAb and spike protein IgG) and air pollution. While the p values for effect estimates are not presented, the presence of non-statistically significant findings can be inferred by the confidence intervals crossing zero.”

*In this context, conducting a multi-center study would provide more valuable insights, benefiting from larger samples and more variability of exposures across participants. Therefore, the possibility of future multi-center studies should be indicated in the Discussion section.*

**Author response:** We agree that expansion of this pilot study is needed for greater insight into the research question. As suggested, we have added the need for a multi-center study to the discussion and conclusion. Lines 345-347 and 366.

*About results:
- Table 3: Insert the information that values refer to the log-transformed neutralizing antibody levels in the second line.*

**Author response:** Thank you for catching this omission. As suggested we have added lo neutralizing antibody levels in the second line of Table 3.  *- I understand that Figures 1 and 2 are suitable for visualizing the effects. However, if I am not mistaken, all information in Figure 1 and Figure 2 is already present in Table 4. If this is really the case, Figure 1 and Figure 2 are redundant and should be removed from the manuscript. In this way, a results description should be in Table 4 accordingly.*

**Author response:** As suggested we have moved the Tables to Supplemental Table S1 so that readers can review the actual data values displayed in the figures if desired. However, we will defer to the editors if Tables are preferred to the figures (and if so figures can be moved to the supplement and tables can be present in manuscript).

*In the abstract and the Discussion, it is said that unexpectedly spike protein IgG increased 14 days after vaccination. Still, evaluations for most COVID-19 vaccines, including the Astra Zeneca vaccine, should consider a period of 14 days to start counting protection according to WHO guidelines. I would not say that this increase in spike protein IgG was unexpected. Therefore, I recommend avoiding such a statement.*

**Author response:** As suggested, we have removed this statement of the unexpected finding from the abstract and discussion.

*Note: I did not receive the supplement indicated in line 167.*

**Author response:** We apologize that the supplement was not included. We have attached the supplement in the revised version.

6. PLOS authors have the option to publish the peer review history of their article ([what does this mean?](https://urldefense.com/v3/__https:/track.editorialmanager.com/CL0/https:*2F*2Fjournals.plos.org*2Fglobalpublichealth*2Fs*2Feditorial-and-peer-review-process*23loc-peer-review-history/1/010f0193f1dcfaa6-da46ce0a-6b1b-4ece-8727-7d7159d78541-000000/DAFupBRWbZkksIVWrfVh2uDxkV8dZ-3n-mqlsd13vBk=190__;JSUlJSUl!!MkQhnT5yHH0MPvOc00c!Xs1LgmE2i8AtIIpO07Q2gZzY9x45TD-QYGT_JVG0V7BVjcqjooKSlkm8qUy_TP285Xc61QCnA-WqUUukKrNXNyi5tQ59$)). If published, this will include your full peer review and any attached files.


**Do you want your identity to be public for this peer review?** If you choose “no”, your identity will remain anonymous but your review may still be made public.


For information about this choice, including consent withdrawal, please see our [Privacy Policy](https://urldefense.com/v3/__https:/track.editorialmanager.com/CL0/https:*2F*2Fwww.plos.org*2Fprivacy-policy/1/010f0193f1dcfaa6-da46ce0a-6b1b-4ece-8727-7d7159d78541-000000/dVyGwPWbzFg-VmdzoH-AX3XDlsT5alAW_h87t2jm69E=190__;JSUl!!MkQhnT5yHH0MPvOc00c!Xs1LgmE2i8AtIIpO07Q2gZzY9x45TD-QYGT_JVG0V7BVjcqjooKSlkm8qUy_TP285Xc61QCnA-WqUUukKrNXNyl5OSRi$).

Reviewer #1: **Yes:** Francesco Forastiere

Reviewer #2: No

[NOTE: If reviewer comments were submitted as an attachment file, they will be attached to this email and accessible via the submission site. Please log into your account, locate the manuscript record, and check for the action link "View Attachments". If this link does not appear, there are no attachment files.]

While revising your submission, please upload your figure files to the Preflight Analysis and Conversion Engine (PACE) digital diagnostic tool, [https://pacev2.apexcovantage.com/](https://urldefense.com/v3/__https:/pacev2.apexcovantage.com/__;!!MkQhnT5yHH0MPvOc00c!Xs1LgmE2i8AtIIpO07Q2gZzY9x45TD-QYGT_JVG0V7BVjcqjooKSlkm8qUy_TP285Xc61QCnA-WqUUukKrNXN9ZtaiUe$). PACE helps ensure that figures meet PLOS requirements. To use PACE, you must first register as a user. Registration is free. Then, login and navigate to the UPLOAD tab, where you will find detailed instructions on how to use the tool. If you encounter any issues or have any questions when using PACE, please email PLOS at [figures@plos.org](mailto:figures@plos.org). Please note that Supporting Information files do not need this step.

*In compliance with data protection regulations, you may request that we remove your personal registration details at any time.* [*(Remove my information/details)*](https://urldefense.com/v3/__https:/track.editorialmanager.com/CL0/https:*2F*2Fwww.editorialmanager.com*2Fpgph*2Flogin.asp*3Fa=r/1/010f0193f1dcfaa6-da46ce0a-6b1b-4ece-8727-7d7159d78541-000000/XabOYXTdUVTCZJVziPaWhTGUxcsTrM19c91sKtRMYuo=190__;JSUlJSU!!MkQhnT5yHH0MPvOc00c!Xs1LgmE2i8AtIIpO07Q2gZzY9x45TD-QYGT_JVG0V7BVjcqjooKSlkm8qUy_TP285Xc61QCnA-WqUUukKrNXN06wfCR0$)*. Please contact the publication office if you have any questions.*
