# Supplementary material for: Ambient air pollution exposure and effects on neutralizing antibody titers following SARS-CoV-2 vaccination in adults
Source: PLOS Glob Public Health. 2025 May 12;5(5):e0004609. doi: 10.1371/journal.pgph.0004609 (PMC12068591; doi:10.1371/journal.pgph.0004609)
Supplement: Attachment — Submitted filename: Response to reviewers_Vaccineairpollution_Croft_2nd rev.docx [file pgph.0004609.s006.docx]

PGPH-D-24-00744
Ambient air pollution exposure and effects on neutralizing antibody titers following SARS-CoV-2 vaccination in adults
PLOS Global Public Health

Dear Colleagues,

Thank you for reviewing our revised manuscript. Please find our responses to your thoughtful comments below. Please note that line numbers refer to the tracked changes version with ‘All markup’ selected.

Sincerely,

Daniel Croft MD, MPH, ATSF

Associate Professor

Dept of Medicine: Pulmonary and Critical Care Division

Dept of Environmental Medicine

University of Rochester Medical Center

*PLOS Global Public Health*

6. Review Comments to the Author

Please use the space provided to explain your answers to the questions above. You may also include additional comments for the author, including concerns about dual publication, research ethics, or publication ethics. (Please upload your review as an attachment if it exceeds 20,000 characters)

Reviewer #2: In the Author Summary, a suggestion is to rephrase in line 53 to: the spike protein, a selected antigen from the SARS-CoV-2 virus.

**Author response: As suggested, this phrasing has been updated**

This sentence requires correction - it seems truncated: “in a simple descriptive sensitivity analysis, we explored whether or not median antibody concentrations different in individuals with or without gas stoves (without modelling ambient air pollution).”

**Author response: As suggested, we have clarified this sentence on lines 264-266**

“In a simple descriptive sensitivity analysis, we explored whether or not median antibody concentrations were different in individuals with or without gas stoves in their homes. For simplicity, we explored this gas stove/median antibody concentration association independent of ambient air pollution.”

In my opinion, the tables are more informative instead of Figures 1 and 2 for the reasons that the authors indicated, i.e., that any reader can review the actual data values from the tables. I highly suggest to have the tables in the main manuscript instead of the Figures.

**Author response:**

As suggested, we have retained the tables in the main document and relabeled the figures to be in the supplement.

Some units are missing from the information in Tables 2, 3, and 5.

**Author response:**

As suggested, the units for the air pollutants were included in the Table 2 and Table S1 (now table 4). For the spike protein, arbitrary units/ml were used so this was added to Tables 3 and 4 (now table 5). Neutralizing Antibody is unitless and no units were included for the log transformed values of spike protein (also unitless).
