# Supplementary material for: Ambient air pollution exposure and effects on neutralizing antibody titers following SARS-CoV-2 vaccination in adults
Source: PLOS Glob Public Health. 2025 May 12;5(5):e0004609. doi: 10.1371/journal.pgph.0004609 (PMC12068591; doi:10.1371/journal.pgph.0004609)
Supplement: Attachment — Submitted filename: Response to reviewers_Vaccineairpollution_Croft_3rd rev.docx [file pgph.0004609.s007.docx]

PGPH-D-24-00744
Ambient air pollution exposure and effects on neutralizing antibody titers following SARS-CoV-2 vaccination in adults
PLOS Global Public Health

Dear Colleagues,

Thank you for reviewing our revised manuscript. Please find figure 1 in the attached article.

Sincerely,

Daniel Croft MD, MPH, ATSF

Associate Professor

Dept of Medicine: Pulmonary and Critical Care Division

Dept of Environmental Medicine

University of Rochester Medical Center

*PLOS Global Public Health*

*Reviewer #2: All comments have been addressed. However, the manuscript contains a reference to Figure 1, which is missing from the submission and was not present in previously submitted versions. I went through the submission file multiple times but could not locate it. Please provide this figure.
"Figure 1: Design of the pilot secondary analysis of a clinical trial, with results."*

**Author response: Apologies that the Figure 1 did not appear in your pdf. Figure 1 should have been present on Page 27 of the pdf composite. We have added the figure to the manuscript and I am hopeful it will appear for your review.**
